# Automated image segmentation uncovers the association of a microglial subtype defined by High CD74 Expression with cognitive decline

**DOI:** 10.1002/alz.092664

**Published:** 2025-01-03

**Authors:** Mariko Taga, Masashi Fujita, Neelang Parghi, Philip L. De Jager

**Affiliations:** ^1^ Columbia University Irving Medical Center, New York, NY USA; ^2^ Center for Translational & Computational Neuroimmunology, New York, NY USA; ^3^ Columbia University Irving Medical Center and the Taub Institute for Research on Alzheimer’s Disease and the Aging Brain, New York, NY USA; ^4^ Center for Translational and Computational Neuroimmunology, Columbia University, New York, NY USA; ^5^ Department of Neurology, College of Physicians and Surgeons, Columbia University and the New York Presbyterian Hospital, New York, NY USA; ^6^ Center for Translational and Computational Neuroimmunology, New York, NY USA; ^7^ Center for Translational & Computational Neuroimmunology, Columbia University Irving Medical Center, New York, NY USA; ^8^ Taub Institute for Research on Alzheimer’s Disease and the Aging Brain, Columbia University Irving Medical Center, New York, NY USA

## Abstract

**Background:**

Stage III activated microglia have been associated with Alzheimer’s Disease (AD) and cognitive decline. Separately, recent single‐cell RNA‐sequencing revealed CD74 as a marker gene that is enriched in immunologically active microglial subtypes associated with AD.

**Method:**

Post mortem tissue sections from the dorsolateral prefrontal cortex were stained simultaneously for (1) CD74, (2) IBA1 (a general microglial marker that outlines cellular processes), and (3) phosphoTau (AT8 antibody) to locate Tau proteinopathy. 154 aged individuals with and without AD from two brain collections were stained: ROS/MAP (n = 63) and NYBB (n = 91). To ensure unbiased results and facilitate high‐throughput data analysis, the entire tissue sections were scanned using a Nikon immunofluorescence scanner. An image analysis pipeline using CellProfiler software has been developed, to automatically delineate the grey and white matter and to extract microglial cell morphology and IBA1/CD74 intensities from a total of 483,733 neocortical microglia.

**Result:**

In a meta‐analysis of the two datasets, the frequency of microglia with CD74 high expression (CD74^high^) is increased in individuals with a clinical diagnosis of AD dementia compared to those without (p = 0.01). These microglia exhibit significantly higher IBA1 intensity (p = 4.1 × 10^‐20^) and adopt a less complex, amoeboid shape (p = 1.1 × 10^‐23^) when compared to the remaining microglia. Results are consistent in samples from each of the contributing brain banks. Since we have a large surface area, we also analyzed the spatial distribution of CD74^high^ cells and note heterogeneity in the distribution of these cells along the cortical ribbon, suggesting that their presence is influenced by regional factors. These topological analyses are ongoing, leveraging the tangle staining.

**Conclusion:**

We report a potential role of CD74^high^ microglial cells in cognitive decline and present evidence that they have a morphologic characteristic that is shared with stage III microglia. Thus, we have used a new automated image segmentation pipeline optimized for the characterization of microglia to define and explore the functional characteristics and topological features of a microglial subtype associated with AD that was originally defined using single cell transcriptomic profiles.

